# Anti-Inflammatory Property of the Ethanol Extract of the Root and Rhizome of *Pogostemon cablin* (Blanco) Benth

**DOI:** 10.1155/2013/434151

**Published:** 2013-12-09

**Authors:** Chu-Wen Li, Xiao-Li Wu, Xiao-Ning Zhao, Zu-Qing Su, Hai-Ming Chen, Xiu-Fen Wang, Xiao-Jun Zhang, Hui-Fang Zeng, Jian-Nan Chen, Yu-Cui Li, Zi-Ren Su

**Affiliations:** ^1^School of Chinese Materia Medica, Guangzhou University of Chinese Medicine, Guangzhou 510006, China; ^2^The First Hospital Affiliated Guangzhou University of Traditional Chinese Medicine, Guangzhou, Guangdong 510405, China

## Abstract

The aim of this study was to investigate the anti-inflammatory property of the ethanol extract of the root and rhizome of *Pogostemon cablin* (ERP). The anti-inflammatory effect was evaluated using four animal models including xylene-induced mouse ear edema, acetic acid-induced mouse vascular permeability, carrageenan-induced mouse pleurisy, and carrageenan-induced mouse hind paw edema. Results indicated that oral administration of ERP (120, 240, and 480 mg/kg) significantly attenuated xylene-induced ear edema, decreased acetic acid-induced capillary permeability, inhibited carrageenan-induced neutrophils recruitment, and reduced carrageenan-induced paw edema, in a dose-dependent manner. Histopathologically, ERP (480 mg/kg) abated inflammatory response of the edema paw. Preliminary mechanism studies demonstrated that ERP decreased the level of MPO and MDA, increased the activities of anti-oxidant enzymes (SOD, GPx, and GRd), attenuated the productions of TNF-*α*, IL-1*β*, IL-6, PGE_2_ and NO, and suppressed the activities of COX-2 and iNOS. This work demonstrates that ERP has considerable anti-inflammatory potential, which provided experimental evidences for the traditional application of the root and rhizome of *Pogostemon cablin* in inflammatory diseases.

## 1. Introduction

Inflammatory reaction, typically characterized by redness, swelling, heat, and pain, is a common physiologic responses of the host to injurious stimuli such as pathogens, toxins, and local injuries [1, 2]. Thus, inflammation has been regarded as a protective attempt to eliminate the harmful stimuli and to activate the healing process by the organism. On the other hand, persistent or overinflammatory response results in damage of tissues and possibly failure of organs [1]. Nowadays, nonsteroidal anti-inflammatory drugs (NSAIDs) are the most commonly prescribed therapeutics for the treatment of inflammatory diseases [3]. However, owing to the adverse side effects such as gastrointestinal ulcers, hemorrhage, and renal damages induced by long-term administration, the use of NSAIDs is becoming highly controversial [3, 4]. Therefore, increasing attention has being focused on the ethnological medicinal plants as they are affordable and with less toxicities and adverse effects [5].

*Pogostemon cablin* (Blanco) Benth. (*P. cablin*), a member of the Lamiaceae family, has been cultivated extensively in China, Korea, Japan, Indonesia, the Philippines, Malaysia, and so on [6]. It has long been used as a traditional medicinal material in China to treat various diseases [7], such as common cold, nausea, diarrhea, headaches, and fever. Nowadays [8], modern pharmacological researches focused more on and laid more emphasis on the dried aerial part of *P. cablin* [9, 10], which also has been defined as the only medicinal and commercial available part of *P. cablin* in China by *China Pharmacopoeia *[8]. However, the root and rhizome of *P. cablin* (RRP) are also important medicinal parts and have been clinically used for treating various inflammation-related diseases in traditional Chinese medicine [11, 12]. There exists few pharmacological research of RRP. Thus, it remains unclear whether RRP possess an anti-inflammatory activity. Therefore, in this work, we aimed to examining the anti-inflammatory activity of the ethanol extract from the root and rhizome of *P. cablin* (ERP).

In this study, we evaluated the anti-inflammatory effect of ERP using four animal models, that is, xylene-induced ear edema, acetic acid-induced vascular permeability, carrageenan-induced pleurisy, and carrageenan-induced hind paw edema in mouse. Furthermore, in order to understand the preliminary anti-inflammatory mechanisms, we determined the levels of inflammatory mediators in the edema tissue of the carrageenan-induced edema paw model, which include tumor necrosis factor-*α* (TNF-*α*), interleukin (IL)-1*β* and IL-6, prostaglandin E_2_ (PGE_2_), nitric oxide (NO), cyclooxygenase-2 (COX-2), and inducible nitric oxide synthase (iNOS). We also evaluated the oxidative stress by quantifying the level of malondialdehyde (MDA), myeloperoxidase (MPO) in the edema tissue, and examining the activity of liver antioxidant enzymes involving superoxide dismutase (SOD), glutathione peroxidase (GPx), and glutathione reductase (GRd). Also, we analyzed the chemical ingredients of ERP using high performance liquid chromatography-photodiode array detector (HPLC-PAD).

## 2. Materials and Methods

### 2.1. Plant Material

The plants of *Pogostemon cablin *(Blanco) Benth (*Labiatae*) were collected from Nursery Garden of South China Medicinal Plants (NGSCMP), Guangzhou University of Chinese Medicine (GZUCM), Guangdong, China, in January 2012 and identified by Professor Lai Xiao-Ping, School of Chinese Materia Medica, GZUCM. The voucher specimen (number VS-2012-1-15B01) was deposited in GZUCM.

### 2.2. Chemicals and Drugs

Carrageenan and Griess reagents were purchased from Sigma-Aldrich (St. Louis, USA). Evans blue, indomethacin, and Tween-80 were purchased from Sinopharm (Shanghai, China). Phosphate-buffered saline (PBS) were purchased from Thermo-Fisher Sci. (MA, USA). Acetonitrile (HPLC-grade) was purchased from Merck (Darmstadt, Germany). All other reagents used were of analytical grade.

### 2.3. Preparation of Plant Extract

Dried roots and rhizomes of *P. cablin* (300 g) were sliced into small pieces and ground into powders following by addition of ethanol (3 L). Then mixtures were ultrasonic extracted at 30°C for 2 h and soakage extracted at room temperature for 20 h. The extraction steps was repeated two additional times, and the extracts were then combined, filtered, and concentrated under reduced pressure at 40°C. A total of 20.46 g of ERP (a yield ratio of 6.82%) was produced. To analyses chemical compositions, ERP was dissolved in 75% ethanol. For pharmacological tests, ERP was dissolved in 0.5% Tween-80 solution.

### 2.4. HPLC-PAD Analysis of ERP

HPLC-PAD analysis were carried out on a Shimadzu LC-20A HPLC system consisting of a SPD-M20A PDA detector, a LC-20AT pump, a SIL-20AC automatic sampler, a CTO-20A thermostatic column compartment, and a Shimadzu LC-20A software (Shimadzu, Kyoto, Japan). The separation was performed on a Phenomenex Synergi Hydro-RP 80A C_18_ column (2.0 × 150 mm, 4 *μ*m, Phenomenex Inc., CA, USA) with a flow rate of 0.3 mL/min, temperature at 30°C, and injection volume of 5 *μ*L. The mobile phase consisted of acetonitrile (A) and 0.1% aqueous formic acid (B) was used with the gradient mode (0–5 min: 5% A→15% A; 5–15 min: 15% A→20% A; 15–20 min: 20% A; 20–30 min: 20% A→30% A; 30–35 min: 30% A→40% A; 35–45 min: 40% A→60% A; 45–55 min: 60% A→95% A; and 55–60 min: 95% A→5% A). The absorption spectra of compounds were recorded from 190 to 800 nm. The analysis based on the retention time and the ultraviolet- (UV-) absorption (190 to 800 nm) of the standard authenticated the presence of verbascoside, rosmarinic acid, and pogostone in ERP. The content of these compounds were quantitative analyzed with peak areas under the standard curves at 254 nm.

### 2.5. Animals

Kunming (KM) mice (20–25 g) were obtained from the Laboratory Animal Services Centre of GZUCM. Animals were maintained on a 12 h light/a 12 h dark cycle under regulated temperature (22 ± 2°C) and humidity (50 ± 10%) and fed with standard diet and clean water *ad libitum*. All studies were conducted in accordance with the National Institutes of Health Guide for the Care and Use of Laboratory Animals. This study used cervical dislocation to sacrifice animals. Except for the acute toxicity study, animals of either sex were divided into 5 groups (10 mice per group, 5 male and 5 female). Positive control indomethacin (10 mg/kg, per animal, p.o.) and ERP (120, 240, and 480 mg/kg, per animal, p.o.) were given. The control group was given an equal volume of vehicle 0.5% Tween-80 solution.

### 2.6. Acute Toxicity Study

In compliance with the criterion previously described [10], KM mice were randomly divided into 3 groups (12 mice per group, 6 male, and 6 female) to evaluate the acute toxicity of ERP after a single oral dose. The mice were administered orally with ERP (1, 2, and 4 g/kg). The experimental mice were provided with forage and water *ad libitum*, and they were kept under regular observation for 14 days for any mortality or behavioral changes. The mortality and behavioral changes include hyperactivity, tremors, ataxia, convulsions, salivation, diarrhea, lethargy, sleep, and coma.

### 2.7. Xylene-Induced Mouse Ear Edema

The ear edema analysis in mice was performed using the method proposed in previous studies [9]. Briefly, 60 min after the administration, each animal received 20 *μ*L of xylene on the anterior and posterior surface of the right ear and left ear was considered as negative control. Another 60 min later, the animals were sacrificed and both ears were sampled with a punch (5 mm diameter) and weighted. The extent of ear edema was evaluated by the weight difference between the right and the left ear biopsies of the same animal.

### 2.8. Acetic Acid-Induced Mouse Vascular Permeability

The acetic acid-induced vascular permeability test with modifications was performed as previously described [13]. Briefly, 60 min after the administration, each animal was intravenously injected 0.5% Evans blue solution at 0.1 mL/10 g body weight followed by an intraperitoneal injection of 0.6% acetic acid at 0.1 mL/10 g body weight. 30 min after acetic acid injection, mice were sacrificed and the peritoneal cavity was washed three times with saline (10 mL). Saline washes were filtered and centrifuged for 10 min at 550 ×g. Supernatants were collected and measured at 590 nm by an ultraviolet-visible spectrophotometry (Shimadzu Co. Ltd., Kyoto, Japan). The optical density (OD) of supernatant was measured.

### 2.9. Carrageenan-Induced Mouse Pleurisy

Pleurisy was induced by carrageenan as previously described [14]. 60 min after the final administration, each mouse was anaesthetized with 10% chloral hydrate and give an injection of 1% freshly prepared carrageenan suspension in normal saline into the pleural cavity (at 0.1 mL/10 g body weight). 4 h after carrageenan injection, each animal was sacrificed and the pleural cavity was washed three times with PBS (2 mL). The neutrophils were suspended in PBS and the total neutrophils count was determined with an optical microscope on coulter counter.

### 2.10. Carrageenan-Induced Mouse Paw Edema Model

#### 2.10.1. Paw Edema Assay

The carrageenan-induced mouse hind paw edema test was conducted based on the method described elsewhere [15]. 60 min after the administration, each animal was given a subcutaneous injection of 25 *μ*L of 1% freshly prepared carrageenan suspension in normal saline into the plantar side of the right hind paw. The paw volume was measured before the injection as the basal volume (*V*_*o*_) and at intervals of 1–6 h after the chemical treatment as the pathological volume (*V*_*i*_) using a MK101 CMP plethysmometer (Muromachi Kikai Co., Ltd). Percentage edema degree of paw edema was calculated using the following formula: % edema degree = (V_*i*_ − V_*o*_)/V_*o*_ × 100. Percentage inhibition of paw edema was presented using the following formula: % inhibition = (Mean edema degree (carrageenan) − Mean edema degree (test))/Mean edema degree (carrageenan) × 100.

To evaluate the effects of ERP on proinflammatory factors and oxidative stress, another set of mice was orally administered with 0.5% Tween-80, indomethacin, or ERP. Four hours after carrageenan treatment, animals were sacrificed, and the right hind paws as well as the whole liver tissues were collected. The right hind paws were immediately placed in cold PBS (1 : 9, v/w) and homogenized by using a Tissue Lyser II high-throughput tissue homogenization system (Qiagen Co. Ltd., Hilden, Germany). The homogenate was incubated on ice for 15 min and centrifuged at 10000 ×g for 15 min at 4°C. Then the supernatants were collected and stored at −80°C for later analysis of MPO, MDA, NO, TNF-*α*, IL-1*β*, IL-6, COX-2, and iNOS. The liver tissue was immediately homogenized in cold PBS (1 : 1, v/w). The homogenate was centrifuged at 10000 ×g for 15 min at 4°C. The supernatants were collected and stored at −80°C for later analysis of antioxidant enzyme (SOD, GPx, and GRd) activities.

#### 2.10.2. NO Assay

The content of nitrite as an indicator of NO production in the supernatant was measured according to the Griess reaction method previously described [16]. Briefly, each supernatant was mixed with the same volume of Griess reagents. After incubation for 10 min at room temperature in dark place, the absorbance was measured at 540 nm using Multiskan GO microplate spectrophotometer (Thermo Fisher Scientific, Waltham, Massachusetts, USA).

#### 2.10.3. TNF-*α*, IL-1*β*, IL-6, and PGE_2_ Assay

The levels of TNF-*α*, IL-1*β*, IL-6, and PGE_2_ in the supernatants were measured using enzyme-linked immune sorbent assay (ELISA) kits (R&D Co. Ltd., Abingdon, UK) according to the manufacturer's instructions. Briefly, diluted standards or samples were added to 96-well plates precoated with affinity purified polyclonal antibody specific for mouse TNF-*α*, IL-1*β*, IL-6, and PGE_2_, respectively. The wells were added with Enzyme-linked polyclonal antibodies and incubated at 37°C for 60 min, followed by final washes for 5 times. The intensities detected at 450 nm was measured after addition of substrate solutions and were proportional to the productions of TNF-*α*, IL-1*β*, IL-6, and PGE_2_.

#### 2.10.4. iNOS and COX-2 Assay

The levels of iNOS and COX-2 in the supernatants were measured using ELISA kits (Cusabio Co. Ltd., WuHan, Hubei, China) according to the manufacturer's instructions. Briefly, processes were the same as that was described in Section 2.10.3.

#### 2.10.5. MPO Assay

The MPO activity was determined according to the method previously described [17]. Each aliquot (5 mL) of supernatant was mixed with PBS (pH 7.4, 15 mL), NaH_2_PO_4_ (0.22 M, pH 5.4, 2 mL), H_2_O_2_ (0.026% (v/v), 2 mL), and tetramethyl benzidine (18 mM in 8% (v/v) aqueous dimethyl formamide, 2 mL). After reacting for 10 min at 37°C following the adding of sodium acetate (1.46 M, pH 3.0, 3 mL), the enzyme activity of each reaction mixture was determined at 620 nm.

#### 2.10.6. MDA Assay

The production of MDA was induced by carrageenan injection and evaluated by the method of thiobarbituric acid reacting substance (TBARS). Briefly, MDA reacted with TBARS at 100°C and formed a red complex TBARS which could be recorded and measured at 532 nm.

#### 2.10.7. Antioxidant Enzymes' Activities Assay

SOD was measured using a commercially available Total Superoxide Dismutase Assay Kit with WST-1 (Beyotime Institute of Biotechnology, Shanghai, China) according to the instruction. Briefly, xanthine and xanthine oxidase (XOD) generated superoxide radicals reacted with 2-(4-iodophenyl)-3-(4-nitrophenol)-5-phenyltetrazolium chloride (I.N.T.) to form a red formazan dye which could be recorded and measured at 532 nm. GPx was measured in accordance with the instruction of Glutathione Peroxidase Assay Kit (Beyotime Institute of Biotechnology, Shanghai, China) by detecting the contents of GRd and Nicotinamide Adenine Dinucleotide Phosphate (NADPH) Oxidation of NADPH into nicotinamide adenine nucleoside phosphate (NADP^+^) results in a decrease in absorbance recorded at 340 nm. GRd was measured according to the Glutathione Reductase Assay Kit's (Beyotime Institute of Biotechnology, Shanghai, China) instruction. In the presence of glutathione (GSSG), GRd oxidizes NADPH into NADP^+^ which would be recorded and measured at 340 nm.

### 2.11. Histological Analysis

Biopsies of right hind paws of mice were collected after the induction with carrageenan for 4 h, as mentioned in Section 2.10.1. Tissue slices were fixed in formalin-acetic acid fixative (10% formalin and 1% acetic acid) for 1 week at room temperature, dehydrated, embedded in paraffin, and sectioned into 4 *μ*m. Tissue sections were stained with hematoxylin and eosin (H&E stain) and examined with a BX60 microscope (Olympus Corporation, Kyoto, Japan). Every 3 to 5 tissue slices were randomly chosen from normal group, vehicle control group, indomethacin, and ERP treated groups. Paw swellings and enlarged cavities of every tissue slice were evaluate (40x and 200x). Images were captured with a Canon Power Shot G10 (Cannon Corporation, Shanghai, China).

### 2.12. Statistical Analysis

All the data were expressed as mean ± S.E.M., and statistical analysis was carried out using one-way analysis of variance (ANOVA), followed by Dunnett's test.

## 3. Results

### 3.1. HPLC Analysis of ERP

**Figure 1:**
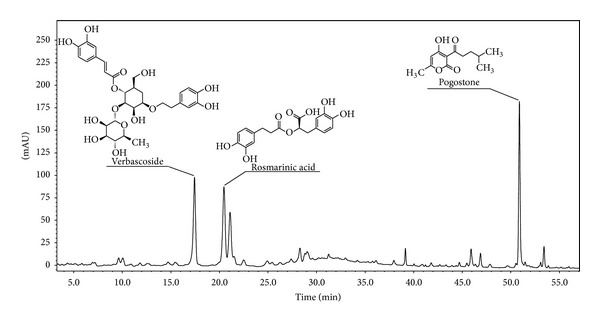
HPLC chromatographs of ERP with detector responses at 254 nm.

The HPLC-PAD analysis profile (254 nm) of ERP was shown in Figure 1, and the relative amounts of three known compounds are verbascoside (26.88 mg/g), rosmarinic acid (28.35 mg/g), and pogostone (46.43 mg/g).

### 3.2. Acute Toxicity Study

After 14 days of administration, ERP did not cause any behavioral changes, and no mortality was observed. Therefore, the LD_50_ value of ERP was concluded to be greater than 4 g/kg in mice, indicating it was practically not acutely toxicity.

### 3.3. Effect of ERP on Xylene-Induced Mouse Ear Edema

**Figure 2:**
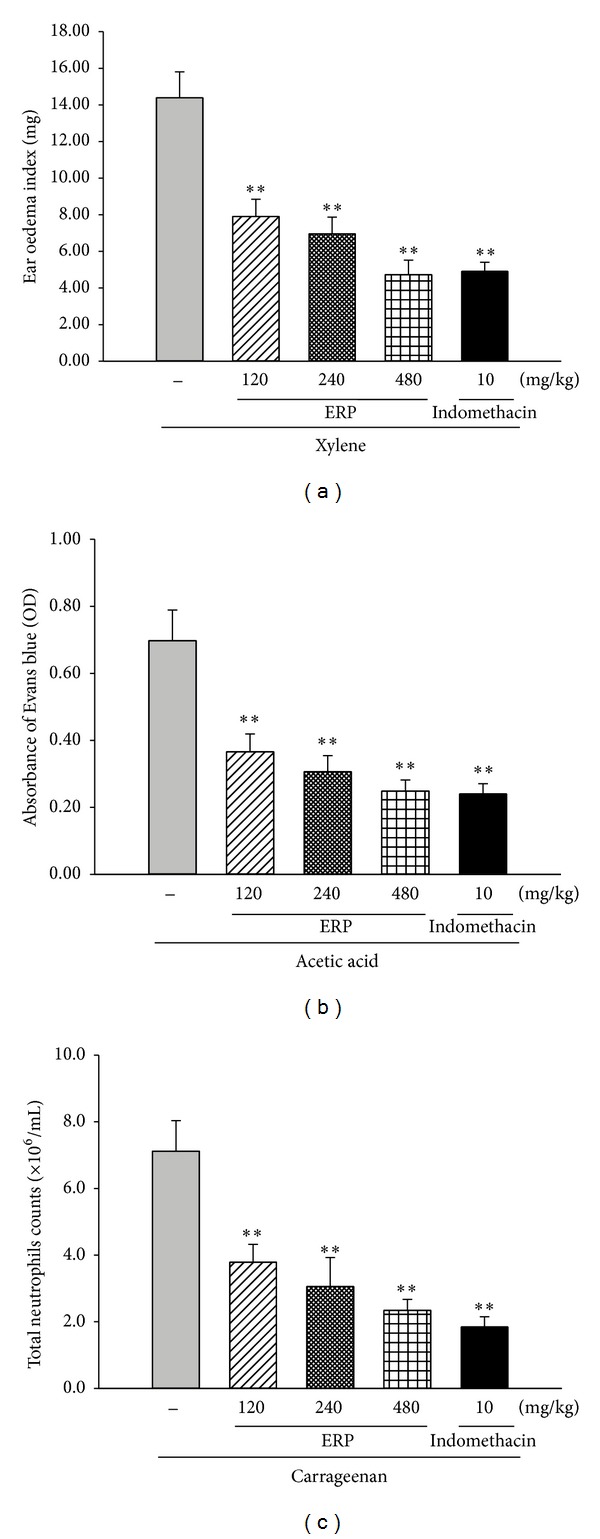
Inhibitory effect of ERP on the xylene-induced mouse ear edema (a), the acetic acid-induced mouse capillary permeability (b) and carrageenan-induced mouse pleurisy (c). (a) The ear edema was represented as the ear edema index which was assessed as the weight difference between the right and the left ear biopsies of the same animal. (b) The capillary permeability was represented by the amount of Evans blue extruded into peritoneal cavity, which was measured by the OD of the supernatant. (c) The pleurisy was represented by neutrophils counts in the inflammatory site. Data was represented as the mean ± S.E.M. (*n* = 10). *P < 0.05 and **P < 0.01 compared to the xylene-control group (a), acetic acid-control group (b) and carrageenan-control group (c).

The ear edema was represented as the ear edema index which was assessed as weight-increase of the right ear over the left one. Application of xylene in mouse ears brought about a significant increase of ear weight in the xylene-control group (Figure 2(a)). In contrast, compared with the xylene-control group, animals in ERP-treated (120, 240, and 480 mg/kg) groups showed significant inhibitions of ear edema at 45.03%, 51.63%, and 67.12%, respectively (for all, *P* < 0.05 versus xylene group). The results demonstrated that oral administration of ERP dose dependently decreased the xylene-induced mouse ear edema. In addition, the inhibition of the group treated with ERP at dose of 480 mg/kg on ear edema was approximate to that of indomethacin group (for both, *P* < 0.01 versus xylene-control group).

### 3.4. Effect of ERP on Acetic Acid-Induced Vascular Permeability

The capillary permeability was represented by the amount of Evans blue extruded into peritoneal cavity, which was measured by the OD value of the supernatant. In current study, the OD value of the acetic acid-control group markedly increased after the challenge of acetic acid (Figure 2(b)). When compared with the acetic acid-control group, treatment of ERP (120, 240, and 480 mg/kg) significantly produced a dose-dependent inhibitory effect on the OD at 47.54%, 56.08%, and 64.42%, respectively (for all, *P* < 0.05 versus acetic-acid-control group). These data showed that ERP markedly attenuated acetic acid-induced capillary permeability.

### 3.5. Effect of ERP on Carrageenan-Induced Mouse Pleurisy

The pleurisy was represented by neutrophils counts in the inflammatory site. Carrageenan injection markedly led to a recruitment of neutrophils in the carrageenan-control group (Figure 2(c)). However, treatment of ERP (120, 240, and 480 mg/kg) showed significant inhibition effect by 46.76%, 57.02%, and 67.13%, respectively (for all, *P* < 0.05 versus carrageenan-control group). These results indicated that neutrophils recruitment induced by carrageenan was suppressed by ERP application.

### 3.6. Effect of ERP on Carrageenan-Induced Mouse Paw Edema

**Figure 3:**
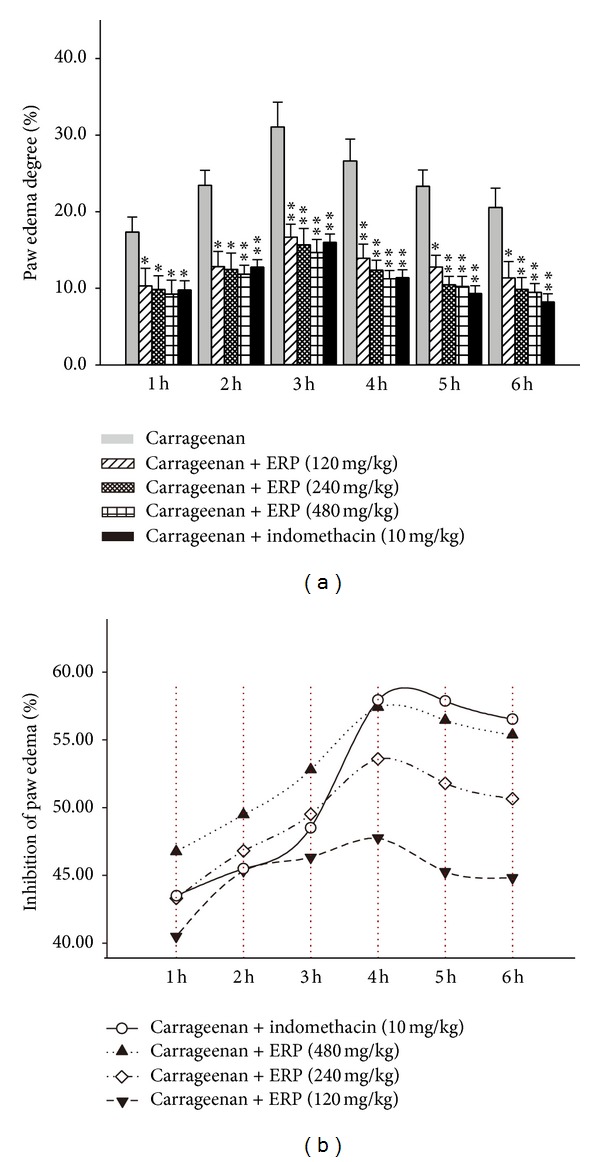
Inhibitory effect of ERP on the carrageenan-induced mouse paw edema. (a) Paw edema degree was represented as the mean ± S.E.M. (*n* = 10). (b) Suppression of paw edema (%) was represented as the ratio of the mean paw size increase of drug treatment group (%) on that of the carrageenan-control group (%). *P < 0.05 and **P < 0.01 compared to the carrageenan-control group.

In this test, the volume of paw edema was measured to evaluate the inflammatory activity. As Figure 3(a) shows, after the carrageenan treatment, the paw volume of the carrageenan-control group obviously increased in a time-dependent manner. When compared with the carrageenan-control group, the ERP (120, 240, and 480 mg/kg) significantly suppressed paw edema increase dose dependently from the 2nd to the 6th hour (for all, *P* < 0.05) after carrageenan treatment (Figure 3(b)). The paw edema inhibition of ERP was peaked approximately at the 4th hour (for all, *P* < 0.05 versus carrageenan-control group) in a dose-dependent manner (Figure 3(b)). Oral administration of ERP at the dose of 400 mg/kg showed almost equal amount of inhibition as 10 mg/kg indomethacin (for both, *P* < 0.01 versus carrageenan-control group) (Figure 3(b)).

#### 3.6.1. Effect of ERP on NO Production and iNOS Ability

**Figure 4:**
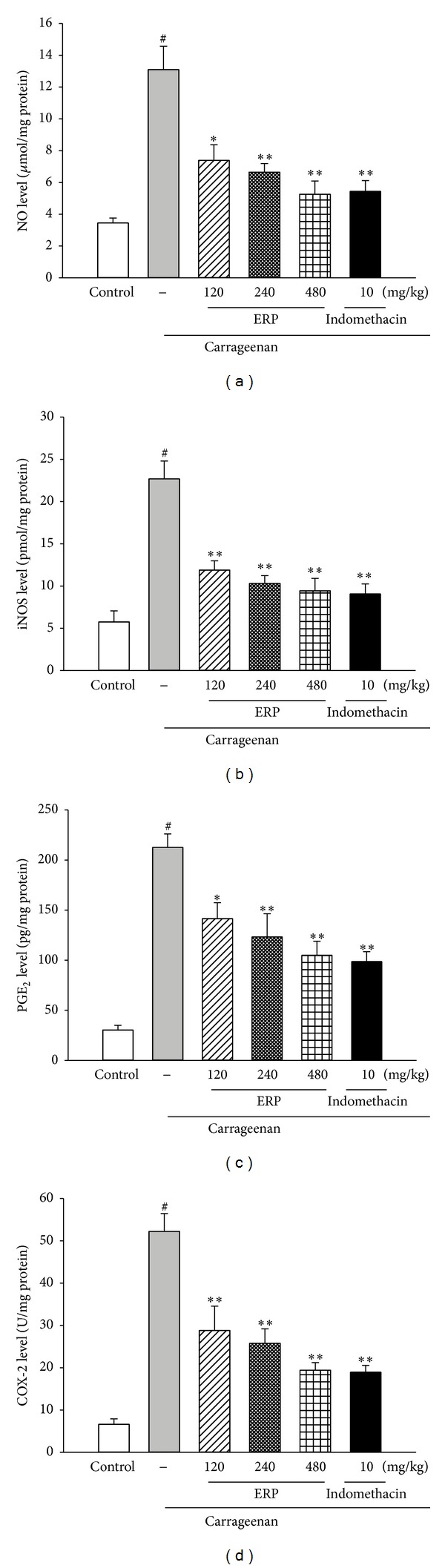
Effect of ERP on carrageenan-induced the level of NO, iNOS, PGE_2_ and COX-2 in mouse paw edema. (a) NO. (b) iNOS. (c) PGE_2_. (d) COX-2. Data represented the mean ± S.E.M. (*n* = 10). ^#^ *P* < 0.01 compared to the control group; *P < 0.05 and **P < 0.01 compared to the carrageenan-control group.

The effect of ERP on the NO production and the iNOS level were shown in Figures 4(a) and 4(b). Carrageenan treatment increased the production of NO and the level of iNOS of the carrageenan-control group (for both *P* < 0.05 versus control group). However, treatment of ERP (120, 240, and 480 mg/kg) significantly decreased the levels of NO and iNOS dose dependently (for all, *P* < 0.05 versus carrageenan-control group).

#### 3.6.2. Effect of ERP on PGE_2_ Production and COX-2 Ability

The effect of ERP on the PGE_2_ production and the COX-2 level were shown in Figures 4(c) and 4(d). Carrageenan treatment increased the levels of PGE_2_ and COX-2 of the carrageenan-control group, compared to the control group (for both *P* < 0.05). However, the levels of NO and iNOS in the ERP (120, 240, and 480 mg/kg) treated groups were significantly suppressed in a dose-dependent manner, as compared to the carrageenan-control group (for all, *P* < 0.05).

#### 3.6.3. Effect of ERP on TNF-*α*, IL-1*β*, and IL-6 Production

When compared with the control, the protein levels of TNF-*α*, IL-1*β*, IL-6, and in carrageenan-induced edema paws of the carrageenan-control group was remarkably raised (Table 1). Oral administration with ERP (120, 240, and 480 mg/kg) had inhibitory effects on TNF-*α* level (both *P* < 0.05). However, ERP at dose of 40 mg/kg did not reduce the TNF-*α* level statistically. In addition, ERP (120, 240, and 480 mg/kg) dose-dependently downregulated the protein level of IL-1*β* and IL-6 as compared to the carrageenan-control group (for all, *P* < 0.05).

#### 3.6.4. Effect of ERP on MPO Activity

**Figure 5:**
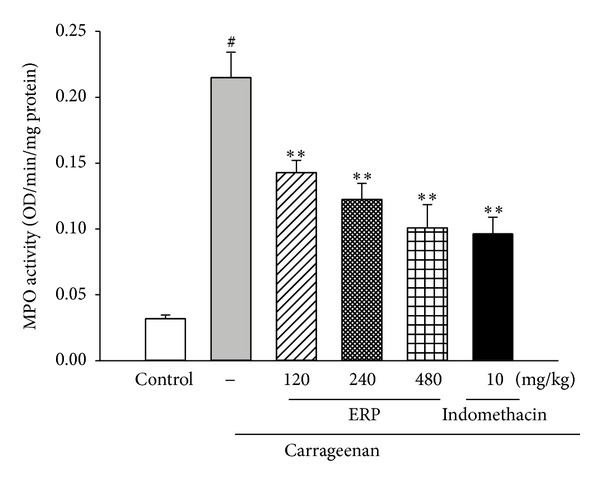
Effect of ERP on carrageenan-induced the activity of MPO in mouse paw edema. Data represented the mean ± S.E.M. (*n* = 10). ^#^ *P* < 0.01 compared to the control group; *P < 0.05 and **P < 0.01 compared to the carrageenan-control group.

As Figure 5 shown, when compared to the control group, the MPO activity of the carrageenan-control group was significantly increased (*P* < 0.01). However, oral administration with ERP (120, 240, and 480 mg/kg) markedly suppressed the MPO activity by 33.55%, 43.01%, and 53.05% (for all *P* < 0.05 versus carrageenan group), respectively.

#### 3.6.5. Effect of ERP on MDA Level

In the carrageenan group, MDA level in the carrageenan-induced edema paw remarkably increased, compared to the control group (Table 2). However, the MDA level decreased significantly in groups treated with ERP (120, 240 and 480 mg/kg) as well as the group treated with 10 mg/kg indomethacin (for all, *P* < 0.05 versus carrageenan-control group).

#### 3.6.6. Effect of ERP on Antioxidant Enzymes' Activities

The activities of SOD, GPx, and GRd at the 4th hour following the intravenous paw injection of carrageenan in mice were decreased significantly compared to the control group (for all *P* < 0.01), as presented in Table 2. However, pretreatments with ERP (120, 240, and 480 mg/kg) boosted the SOD, GPx, and GRd activities significantly (for all, *P* < 0.01 versus carrageenan-control group).

### 3.7. Histopathological Analysis

**Figure 6:**
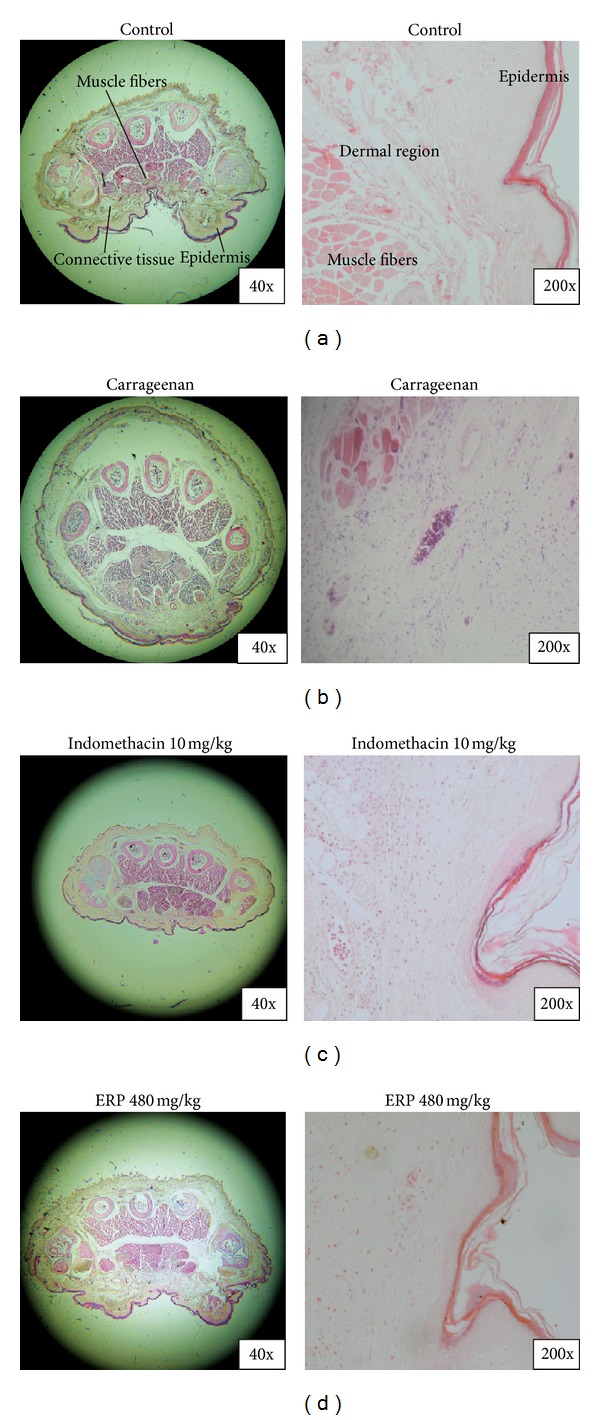
Histopathological examinations on carrageenan-induced paw tissue swelling, edema, haemorrhage, and leukocyte infiltration. (a) Control. (b) Carrageenan-control. (c) Indomethacin (10 mg/kg). (d) ERP (480 mg/kg).

As it is shown in Figure 6(a), no inflammation, tissue destruction, swelling phenomenon, or obvious cellular infiltration was observed in the paws of mice of the control group. On the other hand, the carrageenan-control group displayed enlarged cavities, marked cellular infiltration in the connective tissue, and the infiltrates accumulated between collagen fibres and in intercellular space. (Figure 6(b)). As for positive control group (Figure 6(c)) and experimental group (Figure 6(d)), edematous condition was obviously abated by treatment with indomethacin (10 mg/kg) and ERP (480 mg/kg). When compared to the carrageenan-control group, the collagen fibres of the ERP group were regular in shape; intercellular spaces, inflamed cells, and cellular infiltration were also decreasing.

## 4. Discussion

It is commonly known that inflammation is a complex physiological response and may be acute and chronic [1, 18]. In general, when encountering stimuli acute inflammation, including acute vascular response and acute cellular response, is activated [1, 18]. The acute vascular process results from vasodilation and capillary permeability accentuation and leads to hyperemia and edema formations at the inflammatory site. And the acute cellular one involves the infiltrations of leukocytes, particularly neutrophils, from the blood into the tissue. These processes are all accompanied by cytokine releases, proinflammatory mediator productions, and activations of complement factors [1]. In addition, activated neutrophils secrete multifarious proteases and generate reactive oxygen species (ROS), both of which destroy invading particles but also damage cells and tissues of the host [19, 20]. If acute inflammation is sufficiently severe, it will lead to chronic inflammation. Nevertheless, the excessive acute inflammation can be harmful, even lethal to the host [1, 18]. In this study, xylene-induced ear edema, acetic acid-induced vascular permeability accentuation, carrageenan-induced pleurisy, and carrageenan-induced paw edema in mouse are applied to examining the vasodilatation, vascular permeability, neutrophils infiltration, and edema formation, respectively [18].

During the process of inflammation, vasodilatation brings about plasma extravasations and inflammatory mediators releases, which trigger the acute inflammation response [1]. As we know, xylene-induced mouse ear edema is commonly used to evaluate the levels of vasodilatation and plasma extravasations of neurogenic inflammation [9]. In this assay, treatment with ERP (120–480 mg/kg) significantly decreased the ear edema in a dose-dependent manner. The inhibition of ear edema indicated that ERP attenuated vasodilatations and plasma extravasations of neurogenic inflammation, which were crucial in controlling the early stage of acute inflammation.

In acetic acid-induced vascular permeability test, acetic acid challenge brings about increases in the level of mediators such as prostaglandins, serotonin, and histamine in peritoneal fluids, which in turn lead to a dilation of the capillary vessels and an increase in vascular permeability [13, 18]. Experimental data showed that ERP (120–480 mg/kg) dose dependently attenuated the capillary permeability accentuation induced by acetic acid in mice. Therefore, this result suggested that the anti-inflammatory effect of ERP on the acute phase of inflammation might be associated with prevention of vasodilation and inhibition of the releases of inflammatory mediators.

It is commonly known that there are direct correlations between the role of neutrophils at the inflamed sites and the progress of inflammation-related disorders, and neutrophils play special roles in the pathophysiology of inflammation [21]. Carrageenan treatment results in the recruitment of neutrophils to the inflammatory sites, and carrageenan-induced pleurisy model is well applied in neutrophils migration studies [14]. In current assay, results showed that ERP significantly abolished the rolling and adherence of neutrophils, which led to impairing neutrophils migration from blood vessels into tissues during the acute inflammatory response. Thus, we assume that the anti-inflammatory properties of ERP may probably be related to the inhibition of leukocytes recruitment, especially neutrophils.

The carrageenan-induced mouse paw edema is a reliable and repeatable model for evaluating the anti-inflammatory effect of natural products [22]. Thus, this model was used in our study and the paw edema degree was tested. Oral administration with ERP significantly and effectively inhibited paw edema under challenge of carrageenan in a dose-dependent manner. Consistent with the above results, histopathological examination demonstrated that ERP (at a dose of 480 mg/kg) significantly downregulated the carrageenan-induced inflammatory response in mouse [23]. The considerable anti-inflammatory effects of ERP on the aforementioned animal inflammation models encouraged us to test its anti-inflammatory mechanism. The development of carrageenan-induced paw edema is commonly characterized as a biphasic event. The first phase of edema (0–2 hour) is mediated by histamine and serotonin followed by kinin and finally through bradykinin, PGs, and lysosome [18, 22]. The late phase (2–6 hour) is correlated with the enhanced production of NO, PGs, TNF-*α*, IL-1*β*, and IL-6, which could in turn worsen the level of inflammatory response [13, 18, 22]. TNF-*α*, IL-1*β*, and IL-6 are involved in neutrophil migration in carrageenan-induced inflammation [9, 10, 13, 24]. These mediators are able to recruit leukocytes, such as neutrophils, which were supported by several experimental models in recent reports [18]. In current study, ERP significantly inhibited carrageenan-induced inflammatory response and acted more effectively in the second phase of inflammation than in the first phase. Moreover, the levels of TNF-*α*, IL-1*β*, and IL-6 were also decreased by treating with ERP and indomethacin. Thus, the anti-inflammatory mechanism of ERP may be associated with inhibition on inflammatory mediators, such as TNF-*α*, IL-1*β*, and IL-6.

PGE_2_ serving as the chemotactic and activating factors for inflammatory cells is considered to be one of the most potent modulators in the pathogenesis of various inflammatory diseases [3, 25]. COX-2 is the key enzyme that synthesizes PGE_2_ from arachidonic acid. NO is also a crucial mediator leading to inflammatory response and generated via the oxidation of the terminal guanidine nitrogen atom of L-arginine by iNOS [25]. The NO and iNOS pathway has been reported to contribute to the generation of inflammatory response [3, 25]. Therefore, inhibitions of NO and PGE_2_ production via suppressing iNOS and COX-2 expression are beneficial in treating inflammatory diseases. Our study demonstrated that the ERP downregulated iNOS and COX-2 protein expression, suggesting that the anti-inflammatory effect of ERP might be related to the inhibition on PGE_2_ and NO synthesis. This proposed mechanism is similar to that of indomethacin, the drug used as the positive control, which mediated anti-inflammation via inhibiting PGE_2_ and NO pathways in carrageenan-induced inflammation [26].

MPO is a common *in vivo *index of neutrophil infiltration, inflammation as well as a marker of oxidative stress [17, 27, 28]. Results demonstrated that ERP markedly suppressed MPO activity in inflamed tissues, indicating that both oxidative stress might be alleviate with the treatment of ERP. Therefore, another possible mechanism behind the anti-inflammatory effect of ERP could be attributed to its antioxidative activity.

Previous researchers have also demonstrated that the carrageenan resulted in oxidative stress in inflamed site, which is linked to the production of ROS [27]. ROS such as hydrogen peroxide (H_2_O_2_), superoxide (O_2_^•−^), and hydroxyl radicals (OH^•−^) play major roles in terms of producing cellular damage in inflammatory processes [7]. MDA is produced via free radical attack on the plasma membrane, and it forms covalent protein adducts [27]. Thus, the accumulation of MDA resulting from the inflammatory effect manifests the degree of inflammation. ROS has been proposed to mediate cell damage via a number of independent mechanisms including the inactivation of antioxidant defense systems [7, 27]. A variety of antioxidant enzymes, such as SOD, GPx, and GRd, can scavenge and minimize the formation of ROS [7, 20]. SOD protects cells against the damages of ROS [7, 20]. GPx in the presence of glutathione (GSH) accelerates the reduction of H_2_O_2_ or other organic hydroperoxides and serves as a second line of defense against hydroperoxides [7, 20]. GRd plays a crucial role in cellular defense against oxidative stress by preventing accumulation of oxidized glutathione (GSSG), thus maintaining the redox state [7, 20]. In this study, there was a significant decrease in the level of MDA after ERP treatment, which indicated that inflammation and oxidative stress in carrageenan-induced paw edema were alleviate. On the contrary, ERP significantly enhanced the activities of SOD, GPx, and GRd in the liver. These present results showed that ERP exhibited a positive regulation of antioxidative activities against inflammatory oxidation. Therefore, we assume that the suppressions of MDA production are probably related to the activation of antioxidant enzymes including SOD, GPx, and GRd.

In current study, we quantitatively determined three major components: rosmarinic acid, verbascoside, and pogostone. Rosmarinic is a natural active phenolic ingredient in many Lamiaceae herbs and exhibits significant ROS scavenging, neutrophil infiltration inhibition properties, and it has been reported to be an effective protector against peroxynitrite-mediated damage, and as a potent inhibitor of superoxide and NO synthesis [7, 29, 30]. Previous studies also revealed that rosmarinic acid could dose dependently downregulate proinflammatory mediators, such as TNF-*α*, IL-1*β* and IL-6, and increase the anti-inflammatory cytokine IL-10 [29, 31]. In addition, verbascoside could decrease free radicals concentration and suppress lipid peroxidation [32], which partly contributed to its antioxidative and anti-inflammatory properties [32], and this agent has already been used as possible antioxidants and anti-inflammation in food [32, 33]. Pogostone, a main ingredient of ERP, is also a major constituent of the essential oil of *P. cablin*, which has a variety of pharmacological activities including anti-inflammatory effects [9, 10, 34]. Thus, the chemical analysis of ERP suggested that these major components are possibly responsible for the regulation of inflammatory factors against inflammation response.

In summary, this paper analyzed the chemical composition of ERP, and three compounds: rosmarinic acid, verbascoside, and pogostone were quantified by HPLC-PAD. This study investigated the anti-inflammatory property of ERP using four animal models. The results proved that oral administration of ERP (120, 240, and 480 mg/kg) possessed potent anti-inflammatory, and its mechanism may be related to its antioxidation effect and regulation on inflammatory factors. This study provided experimental evidences for the application of the root and rhizome of *P. cablin* in inflammatory diseases.

## Figures and Tables

**Table 1:** Effect of ERP on carrageenan-induced the levels of TNF-*α*, IL-6 and IL-1*β* in mouse paw edema.

Groups	TNF-*α* (pg/mg protein)	IL-6 (ng/mg protein)	IL-1*β* (ng/mg protein)
Control	5.62 ± 1.72	28.66 ± 3.85	22.36 ± 2.30
Carrageenan	61.11 ± 5.22^#^	188.47 ± 15.69^#^	136.41 ± 11.01^#^
ERP (120 mg/kg)	39.85 ± 4.89*	136.35 ± 13.14*	72.19 ± 8.84*
ERP (240 mg/kg)	33.00 ± 2.79**	124.76 ± 14.80*	66.10 ± 6.52**
ERP (480 mg/kg)	28.44 ± 4.98**	105.18 ± 16.37**	59.67 ± 5.38**
Indomethacin (10 mg/kg)	24.13 ± 3.17**	95.20 ± 10.51**	52.84 ± 7.12**

Data represented the mean ± S.E.M. (*n* = 10). ^#^ *P* < 0.01 compared to the control group; *P < 0.05 and **P < 0.01 compared to the carrageenan-control group.

**Table 2:** Effect of ERP on carrageenan-induced the level of MDA in mouse paw edema and the activities of SOD, GPx, and GRd in mouse liver.

Groups	MDA (nmol/mg protein)	SOD (U/mg protein)	GPx (U/mg protein)	GRd (U/mg protein)
Control	0.24 ± 0.09	10.25 ± 0.36	0.28 ± 0.03	2.37 ± 0.30
Carrageenan	1.39 ± 0.10^#^	3.81 ± 0.20^#^	0.09 ± 0.01^#^	0.42 ± 0.09^#^
ERP (120 mg/kg)	0.59 ± 0.06**	5.96 ± 0.21**	0.15 ± 0.03*	1.31 ± 0.23**
ERP (240 mg/kg)	0.50 ± 0.06**	6.33 ± 0.26**	0.19 ± 0.04*	1.61 ± 0.25**
ERP (480 mg/kg)	0.46 ± 0.04**	6.95 ± 0.33**	0.22 ± 0.05**	1.87 ± 0.20**
Indomethacin (10 mg/kg)	0.48 ± 0.04**	6.48 ± 0.16**	0.19 ± 0.02**	1.62 ± 0.14**

Data represented the mean ± S.E.M. (*n* = 10). ^#^ *P* < 0.01 compared to the control group; *P < 0.05 and **P < 0.01 compared to carrageenan-control group.
